# Considerations on BVD eradication for the Irish livestock industry

**DOI:** 10.1186/2046-0481-64-12

**Published:** 2011-10-03

**Authors:** Damien J Barrett, Simon J More, David A Graham, Joe O'Flaherty, Michael L Doherty, H Michael Gunn

**Affiliations:** 1 Dept Agriculture, Fisheries and Food, Sligo Regional Veterinary Laboratory, Doonally, Sligo, Ireland; 2 Centre for Veterinary Epidemiology and Risk Analysis, UCD School of Veterinary Medicine, University College Dublin, Belfield, Dublin 4, Ireland; 3 Animal Health Ireland (AHI), Main Street, Carrick-on-Shannon, Co. Leitrim, Ireland; 4 UCD School of Veterinary Medicine, University College Dublin, Belfield, Dublin 4, Ireland; 5 Dept Agriculture, Fisheries and Food, Central Veterinary Research Laboratory, Backweston, Celbridge, Co. Kildare, Ireland

## Abstract

Animal Health Ireland has produced clear guidelines for the control of Bovine Viral Diarrhoea (BVD) infection in Irish cattle herds. In the course of developing these guidelines it was clear that a framework for regional and/or national BVD control would be required to increase the uptake of BVD control at farm level and reduce the overall prevalence of the disease. This paper assessed the economic impact of BVD, epidemiological aspects of the disease to its control, models of BVD control, international experiences of BVD control programmes. The technical knowledge and test technology exists to eradicate BVD. Indeed, many countries have successfully and others are embarking on control of the disease. The identification and prompt elimination of PI cattle will form the basis of any control programme. The trade of such animals must be curtailed. Pregnant and potentially pregnant carrying PI foetuses pose a significant threat. International experience indicates systematic, well coordinated programmes have the most success, while voluntary programmes can make good initial progress but ultimately fail. The farming community must buy into any proposed programme, and without their support, failure is likely. To buy into the programme and create such a demand for BVD control, farmers must first be well informed. It is likely that stemming economic loss and improving productivity will be the primary motivator at individual farm level.

## 1. Background

Animal Health Ireland (AHI) is an industry-led, not-for-profit partnership between livestock producers, processors, veterinary surgeons, animal health advisers and government. Its remit includes diseases and conditions of livestock which are endemic in Ireland, but which are not currently subject to regulation and coordinated programs of control.

Bovine Viral Diarrhoea (BVD) emerged as a significant animal health concern in a survey of dairy and beef farmers and from a Delphi study of animal health experts [1]. As a result of these studies, BVD control was prioritised as an objective for AHI. A technical working group (TWG) was convened, which consisted of European specialists in bovine health management, of veterinarians from private practice with a special interest in BVD, as well as veterinarians from the state veterinary laboratory service, the pharmaceutical industry and the animal breeding industry. This group initially developed an externally reviewed guide for controlling BVD at individual farm level, outlining the steps required for an individual farmer to eliminate BVD virus from his herd and prevent further introduction of the infection [2]. In the course of the TWG discussions, it was clear that a framework for regional and/or national BVD control would be required to increase the uptake of BVD control at farm level and reduce the overall prevalence of the disease, which in turn will reduce the risk of re-introduction of the virus to those herds from which the disease has been eliminated.

The purpose of this paper was to inform the debate in the livestock industry on a coordinated nation approach to the control/eradication of BVD in Ireland.

## 2. Significance of the cattle industry to the Irish economy

Agriculture is a very significant contributor to the Irish economy. There are 6.6 million cattle in Ireland and this is made up of one million beef suckler cows and one million dairy cows [3]. There are approximately 128,000 land holdings in Ireland, of which approximately 85,000 breed cattle. The gross agricultural output of the beef and dairy industries were calculated to be €2.55 billion in 2009. The agri-food and drink sector accounts for 9% of Irelands economy-wide GVA or about 16% of total industrial sector output, 10.5% of Ireland's exports and 8.2% of total employment [4]. The agri-food and drink sector accounts for 9% of Irelands economy-wide GVA or about 16% of total industrial sector output, 10.5% of Ireland's exports and 8.2% of total employment. The relative importance of agriculture to the economy has increased since the decline in other parts of the economy.

## 3. Economic impact of BVD

### On-farm impact of BVD

BVD infection in dairy herds can result in reduced milk production, poor reproductive performance, growth retardation, increased susceptibility to other diseases, unthriftiness, early culling and increased mortality among young stock [5]. In disease outbreaks, the costs associated with BVD infection have been estimated to range from €19 to €600 per cow. This observed variation may reflect methodological differences, outbreak severity as well as farm and regional economic conditions. An Irish study calculated the cost of a BVD outbreak in a research dairy herd as €88 per cow [6]. This is acknowledged as a conservative estimate as it does not include costs associated with poor calf health.

The data on the economic impact of BVD in beef herds are more limited. A stochastic economic model of active BVD infection in Scottish beef cattle has calculated the cost of BVD in a suckler herd to be £37 (€40 - €44, depending on exchange rate) per cow per annum [7]. This model was based on a herd of 100 cows, 100 calves and 30 replacement heifers, operated as a single management unit. It was assumed that there was no BVD control in the herd and no re-introduction of infection. However, in a field situation where there is no systematic approach to BVD control, the re-introduction of BVD is always a potential risk. The study attributed these costs to immunosuppression of calves (7%), congenital defects/growth retardation (5%), persistently infected (PI) calves (19%), persistently infected cows and heifers (16%), abortions (9%) and other reproductive loss in cows and heifers (44%). It concluded that control of the disease is financially rewarding and worthy of consideration by individual farmers.

### Implications of BVD eradication

Several Scandinavian countries have successfully eradicated BVD [8-10]. BVD control is well advanced in Austria and Switzerland [11,12]. The World Organisation for Animal Health (OIE) have listed BVD. Within the EU, countries that fail to put control programmes in place may be at a competitive disadvantage when trading and competing with BVD free countries. Eradicating BVD will increase efficiency at producer level by increasing reproductive performance and by increasing the productivity of individual animals [13]. This will increase agricultural output, increasing the supply of produce for consumer. This increased efficiency will also reduce the level of carbon emitted per unit of livestock produce [14].

## 4. Epidemiological factors relevant to the eradication of BVD

Successful eradication requires timely detection and elimination of persistently infected animals (PIs). The control measure must be completely effective in breaking transmission, simple in application and be relatively inexpensive to implement. It is important that the source and spread of the disease is well understood and that it is technically feasible to successfully eradicate the disease [15]. BVD has several characteristics that facilitate its eradication as outlined below.

### Direct animal to animal transmission

Cattle can become persistently infected with the virus if they are infected as unborn calves in the uterus in early pregnancy, and such animals are infected for life. Such PI animals are the main source of BVD virus infection, and excrete BVD virus in all their secretions and excretions through out their lives [16]. Direct contact between PIs and non-infected cattle is the most efficient transmission mechanism in field conditions [17,18]. PI cows invariably give birth to PI calves [19], and it is generally accepted that virus transmission diminishes significantly once PI cattle are removed from herds and typically all animals born after the removal of PIs are seronegative for BVDV.

When a PI animal is born, secondary transmission to naïve animals in the herd occurs quickly [20]. Sero-conversion of all naïve in contact animals occurs over a period of two to five months, depending on contact levels with PI animals [21,22]. BVD serology provides a convenient means of monitoring for BVD re-infection in herds that have achieved BVD freedom.

### Role of transient infection

Viraemia as a result of transient infection in acutely infected animals may last for up to 15 days [23]. Transient infection lasts for a much shorter period than persistent infection, and the level of virus excreted is much lower than that of PI animals [24]. There are reports that transient infection can result in the circulation of virus in herds for prolonged periods, without the presence of a PI animal [25]. However, unidentified persistently infected sources, such as aborted/stillborn calves or calves sold prior to identification, may be the source of infection in such cases [24]. Transient infection in naïve pregnant dams established toward the end of the first trimester in pregnancy give rise to the development of persistently infected calves *in utero*.

### The role of immunity

Transient infection is caused when an immunologically naive non-PI animal is exposed to the BVD virus after birth. Clinical signs can vary considerably from virtually none to severe disease. Once recovered, immunity to further BVD infection develops. Natural immunity at individual animal level to BVDV is of a long duration [24]. Once the infection has been eliminated from the herd and as new animals are born and older animals are replaced, herd immunity diminishes and herd susceptibility to re-infection increases, assuming no seropositive animals have been introduced to the herd.

### Relatively low prevalence of persistently infected animals

**Figure 1:**
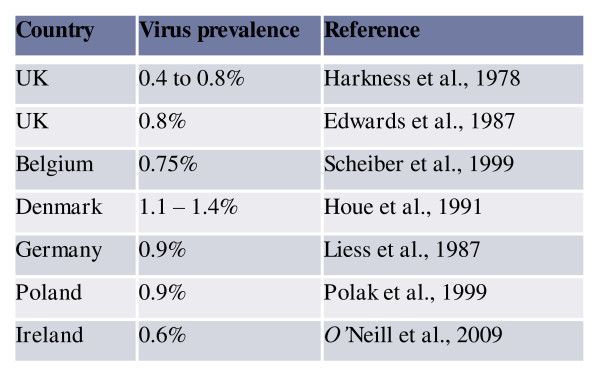
**Prevalence of BVD virus positive animals in a number of European countries cited by (O'Neill et al, 2009)**[14].

A low prevalence of persistently infected (PI) cattle has been described in several European countries [26]; for example, studies have reported a prevalence of PI animals of 0.8% (UK), 0.75% (Belgium), 1.1-1.4% (Denmark), 0.9% (Germany) and 0.9% (Poland). Data from Ireland are broadly in agreement with these figures, where a 0.6% animal level has been reported [26] (Figure 1). The maximum national prevalence of PI is likely not to exceed 2% [27].

### Other methods of animal to animal transmission

Both persistently and transiently infected bulls excrete BVD virus in their semen [28,29]. BVD virus transmission via embryo transfer has been documented, but precautions can be taken to avoid such spread [30]. Transmission between small ruminants and cattle, both ways, has been demonstrated [31-34]. Surveys from Northern Ireland and the Republic of Ireland have recorded individual animal prevalence to Border disease (ovine pestivirus) of 5.3% and 5.6%, respectively [35,36]. Flock-level prevalences of 30.4% and 46%, respectively, were reported in the same studies. Natural transmission of pestivirus from cattle to sheep and vice versa has been documented. However, the pressure of infection is likely to be from cattle to sheep due to the higher herd prevalence of BVD (> 85%), as compared to 30% flock prevalence of pestivirus [35]. Further it was found that the BVD virus was the predominant pestivirus affecting sheep in Northern Ireland [35]. BVDV is the most common contaminant of bovine foetal serum due to the capacity of the virus to spread transplacentally and subsequently establish a persistent infection in the immunologically immature foetus [19].

### Indirect transmission between animals

Indirect transmission of BVD virus by veterinary equipment such as nose tongs, needles [37] and protective plastic gloves worn during rectal palpation [38] have been reported. Flies have also been implicated as a possible indirect transmission route [37]. While these experimental transmission mechanisms are well recognised, their practical importance in relation to between herd transmission cannot be considered as significant as transmission by infected animals. Injectable medicines and vaccines if administered to an infected animal can become contaminated if the same needle is used to withdraw and inject the dose. This can facilitate the spread of infection [39]. It is likely that indirect transmission routes gain increasing relative importance as control programmes advance into later stages [24]. However, as the overall prevalence decreases, so too does the risk of indirect transmission.

### Between-herd spread

The most common method of BVD introduction to a herd is by the purchase of a PI animal or the purchase of a cow carrying a persistently infected foetus [27]. Based on a hypothetical PI prevalence of 2%, the risk of buying a PI was calculated at 33% when buying a group of 20 animals [27]. Even at a relatively low prevalence of PIs, testing animals prior to purchase will reduce the risk of introducing a PI.

Due to the time that often elapses between the introduction of infection and diagnosis of the problem, in many circumstances BVD breakdowns cannot be linked to the purchase of a PI animal. Contact with PI cattle on neighbouring pasture, straying animals, animals returning from shows, markets etc also have a role in the between-herd transmission of BVD. The geographically fragmented nature of Irish cattle farms and their levels of trading with other herds are anecdotally implicated as risk factors for the spread of BVD in Ireland.

BVD virus transmission via embryo transfer has been documented, but precautions can be taken to avoid such spread [30]. BVDV is the most common contaminant of bovine foetal serum (used in embryo transfer and vaccine production) due to the capacity of the virus to spread transplacentally and subsequently establish a persistent infection in the immunologically immature foetus [19].

## 5. General model of BVDV control

BVDV control programmes are based upon the three pillars of (i) biosecurity, (ii) virus elimination and (iii) monitoring [24].

*• Biosecurity: *The emphasis is on preventing the introduction and/or contacts with PI animals and the introduction of dams carrying PI foetuses.

*• Virus elimination: *This applies to infected herds only, where all PI animals are systematically removed.

*• Monitoring: *This involves monitoring the effectiveness of virus elimination in infected herds and detecting new infections in previously BVD-free herds. This is a key step in assessing the overall progress of a control programme.

Vaccination is an optional aspect of control programmes, and can be used in addition to the above in BVD control programmes. For economic reasons, this should be targeted at immunologically naïve animals. Vaccination should never be regarded as a stand-alone control measure, but as an optional measure, in addition to biosecurity, virus elimination and monitoring. It is considered particularly useful in areas of high prevalence, high animal movement and high animal density [40]. However, the potential usefulness of BVD vaccine in systematic BVD eradication programmes has never been assessed.

The purpose of BVD vaccination is to induce an immunity in immunologically naïve animals and thereby reduce the number of susceptible animals, the infectious animal may come in contact with during the course of it infectious period [24], thereby reducing the spread of the disease. However, due to the nature of BVD infection, 100% vaccine efficacy and coverage is required to prevent infection. Several studies have shown that shown the difficulties in achieving such levels of coverage [41-43]. Failure to properly administer the vaccine also reduces the coverage [44]. A further complication of vaccination is that creates a false sense of security among those vaccinating, creating an opportunity for biosecurity breakdowns [45,46].

BVDV control programmes have been classified as systematic and non-systematic [24].

*• Systematic control *involves a widespread reduction in the prevalence of BVD virus and PI animals, typically on a sectoral, regional or national basis. Progress needs to be monitored and evaluated.

*• Non-systematic control *is carried out on an individual herd basis, with no coordinated simultaneous actions in other herds. Vaccination is normally a major component of such programmes.

## 6. International case studies of systematic control

### BVD control in Sweden

The Swedish dairy industry called for the eradication of BVD following the successful eradication of Enzootic Bovine Leucosis, which had been required for entry to the European Union. The Swedish BVD control programme, which began in 1993, was initially voluntary, but it was made compulsory by the industry [8]. The scheme is based on repeated demonstration of the absence of antibodies at herd level, which is required for certification, and a systematic test-and-cull protocol is used for eliminating PI animals from infected herds.

Initially, the programme was funded privately but it has been subsidised by government since its second year. Only animals from BVD free herds can be sold on the open market. However, initially it was possible to sell individual animals tested as being BVD virus free, where the herd was not necessarily free of the disease Those farmers selling animals must have renewed their BVD free status within the previous three months [47]. Compensation for PIs is available from an animal health insurance company, which farmers may subscribe to, to insure against losses caused by animal disease. Improved calf health was the primary motivator among farmers for BVD control and this improvement resulted along with reduced medicine usage and enhanced welfare (Lindberg, personal communication). It proved difficult to motivate those farmers to be interested in regions of Sweden where there was a low prevalence of disease (Lindberg, personal communication). Re-infections into herds that had gained freedom were frequently traced back to non-compliance to scheme rules (Lindberg, personal communication).

The importance of a simple consistent and coherent message from all stakeholders was considered crucial for the success of the programme. Measures perceived to be of importance in successfully progressing the programme were subsidised testing, gradual introduction of regulations to control contacts between herds and prevention of indirect transmission, and eventually making the scheme compulsory [8]. Support from industry stakeholders was considered crucial for the success of the programme, particularly in achieving involvement of all beef herds. One of the key conclusions of the Swedish experience is that if the programme is implemented in a systematic manner and with basic biosecurity and elimination of virus from infected herds and monitoring of non-infected herds, BVDV eradication is possible and profitable [8].

### BVD control in Germany

Prior to 2011, BVD eradication in Germany was voluntary, and practiced to the greatest extent in lower Saxony [40]. Vaccination played a significant role in the programme and was quite unique in that it involved an initial inoculation with an inactivated vaccine followed by a modified live booster, to maximise the immune response [48]. Farmer uptake in Saxony was greatest but nonetheless quite variable. There is considerable evidence from Germany that voluntary schemes, while they may make progress on individual farms, are ultimately unsuccessful, due to variations in motivation, compliance and implementation of the schemes (Moennig, personal communication).

On January 1st 2011, a mandatory BVD control programme was introduced in all German states. The Federal government in Bonn has set baseline requirements for BVD control which all states are obliged to adhere to (Baetza, personal communication). They are free to go beyond the baseline requirements, but must comply with the minimal requirements. The legislation requires all calves to be tested for BVD for the foreseeable future, i.e. the testing is open-ended. However, it is envisaged that this will be amended once the BVD is under control. The legislation requires all PI cattle be destroyed within seven days of confirmation. The movement of PI calves is also prohibited. Direct virus detection is conducted on tissue using ear notch technology using the antigen ELISA or PCR (pooled samples). Samples are collected by farmers at routine tagging, as part of animal registration. Only animals that have been tested as BVD free can be traded, apart from animals that are going to feedlots for slaughter. Herds that are 'unsuspicious' cannot trade with herds that are considered suspicious. There is no Federal (Central) government compensation, however, some states have a semi-state insurance fund, which will compensate farmers for the removal of PI cattle. To receive funds, the farmer involved must make a financial contribution based on the number of animals in his herd (Baetza, personal communication).

### BVD control in Switzerland

The National Bovine Breeders association in Switzerland requested the Federal Veterinary Office (FVO) to commence a BVD control programme in 2004. A major information programme was put in place to convince the various stakeholders, particularly farmers, of the benefits of BVD eradication [49]. This proved to be a critical step, providing information to the cattle industry of BDV related losses and the long opportunities afforded by BVD control. This also helped in gaining acceptance for movement restrictions (particularly of pregnant cattle) following the identification of PI cattle.

The programme was originally planned to start in 2007, but this was postponed until 2008 to ensure it would run smoothly. It was to involve the following steps [12]:

*• Pre-pasturing phase *(Jan to July 2008)

All young bovine animals tested before being moved to summer pastures. Cattle from several herds are co-grazed on the summer pastures. Therefore it was crucial that all animals entering such pastures were known to be BVD free to prevent the spread of infection.

*• Initial phase *(October to December 2008)

All cattle not yet tested were now to be tested with the exception of farms that have only cattle destined for direct slaughter (fattening farms).

*• Calf phase *(January 2009 to September 2009)

After the initial phase, all newborn calves were tested. This was done by the farmer taking an ear tissue sample at tagging.

*• Surveillance phase *(From October 2009 to 2011)

At this stage, the majority of the Swiss cattle population will be tested BVD free (no infected animals on the farm). The purpose of this phase is to verify herds remain free, and to rapidly detect any breakdowns.

The prevalence of PI calves at birth has fallen from 1.5% in 2008 to less than 0.2% in 2010 [49]. The integrated database known as ISVet (animal health database) receives data feeds from both the animal registration and movement database and the laboratory database [49]. This access to movement and laboratory information has been considered crucial in making progress in the Swiss programme.

The Swiss programme is centrally coordinated by the FVO and administered locally by cantonal governments. Such programmes need to be led by a motivated and dynamic person who is sufficiently senior in the organisation to make the necessary decisions quickly to change the programme to improve delivery where necessary. Farmers are paid compensation for the removal of PI cattle (300 CHF per persistently infected animal).

Herd size, early death rate (i.e. the number of animals that either die before 15 days of age or are stillborn per number of newborns per year), buying-in stock, using communal summer grazing, production type, age structure and management strategy were factors associated with the appearance of new cases of infection [49].

### BVD eradication in Scotland

BVD was successfully eradicated from the Shetland Islands in the mid 1990s, using the Scandinavian approach [50]. Unfortunately, it was reintroduced to the islands through purchase of a PI animal, highlighting the need for a monitoring element to such eradication programmes. A voluntary scheme commenced on the Orkney Islands, but despite good initial progress this has stalled, due to incomplete participation by the farming population [51]. The programme was based on the Cattle Health Certification Standards (UK) (CHeCS) programme, which evolved as an independent UK industry body and has provided guidelines for the control and eradication of BVDV, among other infectious cattle diseases, for over 10 years.

Voluntary BVD eradication programmes operated under the auspices of CHeCS have been available in Scotland for several years. The Scottish Government launched a consultation process on BVD control in 2010 and published its plans on eradicating BVD in September 2010 [52]. This will be carried out in three phases:

*• Phase One*. From 22 September 2010 until 31 March 2011, financial support is available for testing for breeding herds and an awareness programme.

*• Phase Two*. From September 2011, all cattle herds in Scotland will be required to be monitored annually.

*• Phase Three*. From September 2012, all identified PI cattle will be required to be slaughtered or housed in secure facilities.

A further phase, which would see movement restrictions placed on herds that failed to tackle their BVD problem, would be introduced at a later date if it was warranted by the disease situation at that point.

## 7. Discussion

BVD is considered by both farmers and experts in animal health as one of the most significant infectious agents affecting Irish cattle [1]. The technical knowledge and test technology exists to eradicate BVD in a cost-effective way. Indeed, many countries have successfully controlled the disease. Therefore, should Ireland decide to embark on a BVD control programme once both the technical knowledge and test technology required can be made widely available.

Various international serological surveys have reported animal level BVD antibody prevalences between 60 and 70%. An Irish survey reported a 78% BVD seroprevalence [53]. An Irish study involving samples submitted for diagnostic purposes calculated a seroprevalence of 69% [26]. Further examination of the data in that study suggested that 50% of Irish herds had evidence of current or recent infection. While this is a convenience sample, and therefore is at risk of bias, a similar level (40%) of Swedish herds had evidence of active or recent infection at the start of their eradication programmes [8]. A herd-based study of randomly selected Irish herds found a herd-level seroprevalence of 98.7% [54]. There was no significant difference between dairy and beef herds (98.5% versus 98.8%, respectively).

Various studies from Europe have estimated the prevalence of BVD virus positive animals to be between 0.75% and 1.4% [26]. Again, data from Ireland are broadly in agreement with these figures, where a 0.6% animal level has been reported in a convenience sample [26] (Figure 1). The maximum national prevalence of PI cattle is not likely to exceed 2% [27]. In other words, the prevalence of BVD at the herd and individual animal levels in Ireland is unlikely to be greatly different from that of many countries with intensive livestock production when their eradication programmes commenced.

The main source of BVD virus infection is PI animals, which are known to excrete large volumes of virus throughout their lives [16]. Direct contact between PIs and non-infected cattle is the most efficient transmission mechanism in field conditions [18]. All other methods of virus transmission, including transiently infected cattle and fomites are considered to require the initial presence of a PI animal. Therefore, PI cattle are crucial for the continuation of the infection in the population, and eliminating these cattle from the population as quickly and as efficiently as possible is the foundation of any BVD eradication programme.

As a population moves, over time, towards BVD freedom, it is important from an epidemiological perspective that PI animals are removed at as young an age as is possible. It is more cost effective for farmers to remove PI calves early in life when their value is less than a more mature animal, thereby minimising both the investment in the animal and the amount of virus it can transmit to cohorts. Although compensation is paid in some countries, it is very much below the market value of a non-PI comrade. Other countries do not pay compensation but put the resources into laboratory testing.

Controlling the trade of PI cattle is crucial. International experience indicates that a prohibition on the sale of PI cattle and pregnant cattle from infected herds must ultimately be put in place to address the herd-to-herd spread of BVD [7,41]. In the Irish context, this would require legislation to prohibit the movement of PI cattle and possibly pregnant cattle from herds where infection has been confirmed.

An intensive, relatively short-term campaign is preferable to a more protracted campaign, where momentum is likely to be lost and enthusiasm and cooperation likely to wane. Protracted campaigns are more likely to allow the virus to re-emerge in herds where the virus has been previously cleared. The identification and prompt elimination of PI cattle will form the basis of any control programme. The trade of such animals must be curtailed. Pregnant and potentially pregnant carrying PI foetuses pose a significant threat [9].

In voluntary programmes, such as those operating in Lower Saxony, uptake varied considerably between farms and regions. There can be considerable differences in their levels of motivation and interest and therefore considerable differences in application and uptake. No matter how committed a farmer is, he/she may have neighbours and be trading with others who are not participating in BVD control programmes [51]. It is the view of German experts that voluntary programmes are ultimately unsuccessful, in spite of the resources put into them. For these reasons, the proposed German programme commencing in January 2011 will be mandatory for all herds. International experience indicates systematic, well coordinated programmes have the best chance of success. In a pan-European study of attitudes toward BVD control and eradication, respondents from countries with voluntary control efforts cited low farmer motivation as a major hindrance to controlling the disease [55]. In the same study, respondents identified the need for a systematic, well coordinated approach as crucial for programme success.

### Infection status of herds

A baseline survey of dairy herds using bulk antibody tests was carried out in Sweden six months prior to the launch of the programme (Lindberg, personal communication). This served a number of purposes. Firstly, it gave an overall disease prevalence for the country, and this in itself is useful information from the point of view of planning the programme. It categorised herds into those likely to be free of disease, and herds likely to have current or recent infection. In this way, resources could be targeted at those herds more likely to be infected and those herds that are likely to be free of disease could be advised on remaining free of disease.

### Farm buy in/farmer demand

In most, if not all, countries where a successful BVD programme is in operation, it has come about as a result of farmer or farmer organisation demand for such an initiative. When the majority of farmers support such initiatives, this can be used to convince farmers who are less interested in participating. In Switzerland, prior to the programme commencing, a farmer information campaign was launched [49]. Farmers in 25 of the 26 cantons (regions) voted to eradicate BVD. This mandate gave the eradication programme authority and authenticity.

The farming community must buy into any proposed programme, and without their support failure is likely [56]. Such support will create a driving momentum and apply peer pressure in the industry to increase uptake and compliance.

### Motivation of farmers to control BVD

Reducing the economic impact and the negative welfare implications of BVD infections at farm level has been the primary motivator for dealing with BVD in the various countries where control programmes have been instigated. It is difficult to maintain farmer enthusiasm with prolonged eradication programmes. This creates a sense of fatigue and reduces enthusiasm. Ironically, this fatigue is often a bigger problem in areas with low disease prevalence as people assume the disease no longer poses a threat leading to complacency (Lindberg, personal communication). To avoid such fatigue and complacency, the programme must initially set achievable targets, realistic time lines and communicate the plan to the farming community. Progress needs to be reviewed frequently with regular updates for farmers. Any barriers to progress need to be identified and addressed at the earliest opportunity.

### Information and raising awareness among farmers

For this demand to come about, farm opinion leaders and the general farming community need to have a good understanding of the clinical consequences and epidemiology of BVD. International experience indicates that farmers must receive a simple coherent message from the various service providers for the information campaign to succeed [56,49]. In practice, this means the various stakeholders agree on the message and farmers receive this single message from all players in the eradication programme. In an online survey of BVD experts, private veterinary practitioners, the farming press and farm advisers were considered the three most effectives conduits for increasing farmers' awareness and understanding of BVD (Barrett, unpublished). These groups play a fundamental role in propagating the information and awareness campaigns.

### Motivation of wider industry and society to control BVD

Enhancing profitability at farm level brings benefits for the entire industry and wider economy. Reducing the clinical manifestations of BVD improves animal welfare and has public health benefits by way of reduced antimicrobial usage [5]. These factors have the potential to add value to livestock produce. Eradicating BVD from the national herd will increase the production efficiency of animals through improved health and fertility. This will have positive implications for greenhouse gas emissions [14]. Adding value and increasing efficiency in this way will aid the livestock industry to achieve the targets set out in the 2020 Food Harvest Strategy [57]. Social and political factors must be considered in attempting to tackle BVD on a national level [55].

Trade between Northern Ireland and the Republic of Ireland is one such political factor. In 2009, 12,700 were imported into Ireland, with half of these coming from the UK. It is likely the majority of these UK animals came from Northern Ireland. In the same year, almost 80,000 animals were exported to Northern Ireland. The land border creates a difficulty in addressing BVD control. Stakeholders on both sides of the border are considering BVD control. However, should one country decide to move forward on BVD control, then animals being traded would need to be tested free of BVD. Controls already in place for TB and brucellosis could be built upon to facilitate the trade continuing.

### The need for central coordination including a central database

It is important that the coordination involves a central database, where movement data and laboratory data are integrated promptly [12]. Any proposed control programme, whether it is a voluntary programme for individual farmers or a full-scale national eradication, needs to be centrally coordinated. This helps to ensure that the programme for each herd is consistently adopted by all participating farmers. It is especially important that problems are identified early so that they may be corrected as early as possible. Both the Irish Cattle Breeding Federation's database and the Department of Agriculture's Animal Health Computer System (AHCS) offer the potential to provide such a database for the Irish cattle industry. Both databases are integrated with the national registration and movement database (AIM). Currently the AHCS database is installed in the vast majority of veterinary practices and is used for TB and brucellosis testing. There is scope to add further diseases to the database. The ICBF database is predominately a breeding and genetics database, but has the potential to integrate disease data.

### Diagnostic testing

Diagnostic testing for BVD is currently based on antibody and virus detection. The Scandinavian countries used antibody tests on individual animals and followed up negative results with virus detection. The test technology has moved on since the initial Scandinavian programmes. The antibody test is the test of choice for surveillance in herds once disease freedom has been achieved. It can also be used to target resources to the herds with current or recent infection. Modern virus detection techniques are much more cost effective than heretofore. These improvements in test technology means that large-scale programmes based on virus detection are much more feasible than heretofore. However, combining virus isolation tests with antibody serology in juvenile animals (6-12 months of age) and bulk milk antibody screening in dairy herds can be used to monitor the effectiveness of the virus elimination tests and increase the overall sensitivity of the programme. This is particularly relevant in identifying any herds where the virus may have been missed.

### Demographic factors

Ireland has one of the largest cattle population densities in Europe. Cattle spend most of the year at pasture. Irish farms tend to be relatively fragmented holding, resulting in boundaries with several neighbours. Serological surveys suggest a relatively high prevalence of infection [26,54]. It has been suggested that this should influence the choice of control strategy [40]. However, experiences from controlling BVD in high-density areas like Denmark and South East Sweden show that herd size, herd density and initial prevalence are not meaningful predictors for the prospects for successful reduction in incidence and prevalence or for the risk of re-infection after virus elimination, not even on the duration of the eradication. Rather, it is the way in which control activities are organized and implemented that will determine the progress [24]. This was clearly demonstrated in Denmark and Norway, where the initial prevalence of dairy herds with recent or ongoing infection in the countries was 40 and 9% respectively, but where both countries will have finalized the eradication after approximately 11 years of activity [58,59].

## 8. Conclusions

BVD is considered by farmers and veterinary surgeons as one of the most significant infectious diseases affecting cattle. The technical knowledge and test technology exists to eradicate BVD. Indeed, many countries have successfully and others are embarking on control of the disease. In many of these countries, livestock production is not as significant to the economy as it is in Ireland. The prevalence of BVD in Ireland is not likely to be much greater than most of the countries which have embarked on BVD eradication.

The identification and prompt elimination of PI cattle will form the basis of any control programme. The trade of such animals must be curtailed. Pregnant and potentially pregnant cattle carrying PI foetuses pose a significant threat.

International experience indicates systematic, well coordinated programmes have the most success, while voluntary programmes can make good initial progress they ultimately fail.

The farming community must buy into any proposed programme, and without their support failure is likely. Farm organisations both here in Ireland and in Scotland have indicated that a sizeable majority of farmers must be supportive for the programme to succeed. Such support will create a driving momentum and apply peer pressure in the industry to increase uptake and compliance.

To buy into the programme and create such a demand for BVD control, farmers must first be well informed. International experience indicates private veterinary surgeons, the farming press and farm advisers are the most influential persuaders of the farming community. However, these groups must first be won over and they must be imparting a simple consistent message.

It is likely that stemming economic loss and improving productivity will be the primary motivator at individual farm level. Improved animal welfare, protecting public health in the context of reduced medicine usage, improved quality assurance enhancing product marketability and increased farm efficiency leading to reduced greenhouse gas emissions are also potential motivators for the wider industry and society in general.

For such a programme to be successful it must be systematically coordinated. International experience indicates that an aggressive short to medium programme is likely to bring the most success. Protracted campaigns lead to disease control fatigue as awareness and motivation wanes, leading to complacency and cynicism. They also afford the BVD virus an opportunity to re-emerge. Such an approach will require laboratory, database and human resources as well as industry buy in and legislative support.

## Competing interests

The authors declare that they have no competing interests.

## Authors' contributions

DB did the literature review, interviews and wrote the manuscript. JOF commissioned the study. MG, DG, SM, JOF and MD provided advice to the lead author. All authors reviewed the manuscript. All authors read and approved the final manuscript.
